# Relation of Baseline Neutrophil Gelatinase-Associated Lipocalin (NGAL) Levels and Contrast-Induced Nephropathy following Percutaneous Coronary Intervention among Chronic Kidney Disease Patients

**DOI:** 10.3390/jcm10225403

**Published:** 2021-11-19

**Authors:** Lior Lupu, Hytham Abukatash, Ariel Banai, Keren-Lee Rozenfeld, Dana Lewit, Ilan Merdler, Itamar Loewenstein, Gil Bornstein, Shmuel Banai, Yacov Shacham

**Affiliations:** Departments of Cardiology& Internal Medicine “B”, Tel-Aviv Sourasky Medical Center Affiliated to the Sackler Faculty of Medicine, Tel-Aviv University, Tel-Aviv 64239, Israel; liorlupu@gmail.com (L.L.); haythamkat@gmail.com (H.A.); arielbanai@gmail.com (A.B.); kerenlees@gmail.com (K.-L.R.); levit.dana@gmail.com (D.L.); Ilanmerdler@gmail.com (I.M.); italoewe@gmail.com (I.L.); gilbo@tlvmc.gov.il (G.B.); shmuelb@tlvmc.gov.il (S.B.)

**Keywords:** neutrophil gelatinase-associated lipocalin (NGAL), acute kidney injury (AKI), chronic kidney disease (CKD), ST-elevation myocardial infarction (STEMI)

## Abstract

Background: The risk of contrast-induced acute kidney injury (CI-AKI) following coronary intervention is particularly high among patients with chronic kidney disease (CKD). Among these patients, baseline neutrophil gelatinase-associated lipocalin (NGAL), a marker of tubular damage, reflects the severity of renal impairment. We evaluated whether the baseline serum NGAL level may be a marker for the development of CI-AKI following percutaneous coronary intervention (PCI). Methods: Eighty-eight CKD patients treated with PCI were included. Serum NGAL levels were drawn upon hospital admission. Receiver operator characteristic (ROC) methods were used to identify the optimal sensitivity and specificity for the observed NGAL level compared with the estimated glomerular filtration rate (eGFR) calculated for patients with CI-AKI. Results: Overall CI-AKI incidence was 43%. Baseline serum NGAL levels were significantly higher in patients with CI-AKI than in patients without CI-AKI (150 vs. 103 ng/mL, *p* < 0.001). According to the ROC curve, baseline NGAL levels performed better than eGFR to predict CI-AKI (AUC 0.753 vs. 0.604), with the optimal cutoff value for baseline NGAL to predict CI-AKI being 127 ng/mL (sensitivity of 68% and specificity of 68%, *p* < 0.001). In a multivariate logistic regression model, the NGAL level >127 ng/mL ng/mL was independently associated with CI-AKI (HR 9.84, 95% CI: 1.96–40.3; *p* = 0.01). Conclusion: Baseline serum NGAL levels in CKD patients may identify a high-risk population for CI-AKI following PCI. Further studies on larger populations are required to validate the potential utility of NGAL measurements in monitoring specific CKD-associated conditions.

## 1. Introduction

Contrast-induced acute kidney injury (CI-AKI) is a common complication among patients undergoing percutaneous coronary intervention (PCI). Neutrophil gelatinase-associated lipocalin (NGAL) is a glycoprotein stored in granules of mature neutrophils released by renal tubular cells following acute tubular damage [1,2,3]. In response to renal tubular injury, the expression of NGAL increases substantially. Elevated NGAL levels can be detected within a few hours following tubular insult and were found to predict earlier renal injury better than serum creatinine (sCr) [4,5]. These findings were demonstrated in various patient populations, including post-cardiac surgery, contrast administration, and septic shock [6,7,8,9]. Recent studies have also reported altered NGAL levels in chronic kidney disease (CKD) patients [10,11,12], suggesting the possibility that NGAL excretion from tubular cells may also reflect active renal damage that underlies the chronic impairment condition. Chronic renal impairment is among the most important risk factors for developing CI-AKI, showing as a symptom in nearly 50% of patients with CKD stage 3/4 [13,14]. To the best of our knowledge, no study to date has investigated the relationship between baseline serum NGAL in CKD patients and the occurrence of CI-AKI. Accordingly, we assessed the predictive ability of serum NGAL to predict acute deteriorations in renal function in CKD patients undergoing PCI.

## 2. Materials and Methods

### 2.1. Patients

We performed a prospective, observational, open-label trial at the Tel Aviv Medical Center. We included patients with CKD admitted to the cardiology ward following PCI between December 2018 and March 2020. The presence of baseline CKD was determined based on past laboratory data and medical reports, as well as admission serum creatinine, and was available for all included patients. Based on the availability of NGAL kits, the study population consisted of 88 patients undergoing PCI. Iodixanol was used as a contrast agent (Visipaque, GE healthcare, Ireland). Physiologic normal saline (0.9%) was administered intravenously at a rate of 1 mL/kg/h for 12 h following exposure to contrast material, although for patients with overt heart failure The hydration rate was reduced for patients with overt heart failure at the physicians’ discretion. The study protocol was approved by the local institutional ethics committee (Institutional Board Review number TLCV-16-224).

### 2.2. Laboratory

Serum samples for baseline NGAL were collected within 12 h following hospital admission. Samples were centrifuged within 10 min of collection using a cooled centrifuge, and plasma and serum were stored at −20 °C. NGAL levels were analyzed using NGAL rapid turbidimetric immunoassay (Bioporto Diagnostics, Copenhagen, Denmark).

Baseline venous blood samples were used for sCr and high sensitivity C-reactive protein (CRP) level measurements. sCr was also tested repeatedly on a daily basis during hospitalization for all included patients. CRP levels were analyzed quantitatively using the Bayer wide-range assay. The estimated glomerular filtration rate (eGFR) was calculated using the Chronic Kidney Disease Epidemiology Collaboration (CKD-EPI) equation [15]. CKD was defined by an eGFR <60 mL/min/1.73 m^2^ based on past laboratory reports, admission blood samples, and patient data. CI-AKI was defined using the KDIGO criteria as either: an increase in sCr by 0.3 mg/dL or more within 48 h following PCI or an increase in sCr above 1.5 times the baseline within seven days of PCI [16].

### 2.3. Statistics

Categorical variables were expressed as frequencies and percentages. The distribution of continuous variables was assessed using histograms and quantile–quantile plots. Normally distributed continuous variables were displayed as means (± standard deviations), and non-normally distributed variables were displayed using median and interquartile range (IQR). Fisher’s exact test was used to evaluate the association between categorical variables. Continuous variables were compared using the independent sample *t*-test for normally distributed data and the Kruskal–Wallis test for non-normally distributed variables. Spearman’s rank correlation was used to measure the correlation between the logarithmic transformations of NGAL and eGFR. Receiver operator characteristic (ROC) curve analysis was performed to identify the optimal cutoff point of serum NGAL levels and eGFR (at which sensitivity and specificity would be maximal) for the prediction of CI-AKI. The area under the curve (AUC) was calculated as a measure of the accuracy of the tests. A multivariate logistic regression model was used to assess the association between serum NGAL levels and AKI, as well as control potential confounders. Initially, univariate regression was employed, and variables with a *p*-value < 0.1 were included in the multivariate binary logistic regression model. A two-tailed *p*-value < 0.05 was considered significant for all analyses. All statistical analysis was performed using R statistical software (version 4.0.5; R Foundation for Statistical Computing, Vienna, Austria).

## 3. Results

**Figure 1:**
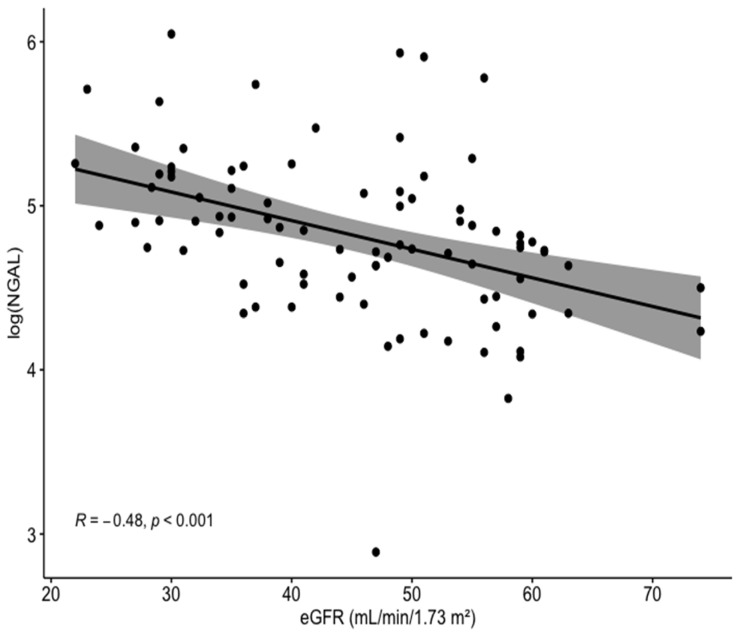
Correlation between the log of baseline serum NGAL levels and eGFR for the entire cohort. NGAL, neutrophil gelatinase-associated lipocalin; eGFR, estimated glomerular filtration rate.

The study included 88 CKD patients treated with PCI (mean age 77, 82% male), 38 (43%) of whom developed CI-AKI throughout hospitalization. Baseline characteristics and laboratory results for CKD patients with vs. without CI-AKI are presented in Table 1. Comparing these two groups, there were no statistically significant differences in age, gender, and co-morbidities. Importantly, no difference was demonstrated between the groups regarding baseline eGFR or the amount of contrast media used during PCI. Interestingly, baseline NGAL levels were significantly higher in patients with vs. without CI-AKI (150 vs. 103 ng/mL, *p* < 0.001). Serum NGAL was significantly and inversely correlated with eGFR (R = −0.48, *p* < 0.001; Figure 1).

**Figure 2:**
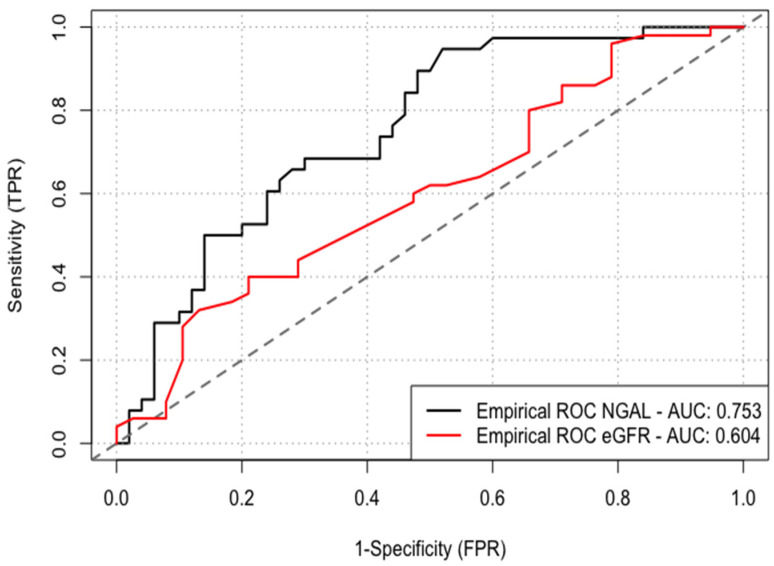
ROC curve analysis was performed to identify the optimal cutoff point of serum NGAL levels and eGFR for the prediction of CI-AKI. NGAL, neutrophil gelatinase-associated lipocalin; eGFR, estimated glomerular filtration rate; CI-AKI, contrast-induced acute kidney injury.

According to the ROC curve, baseline NGAL levels performed better than eGFR to predict CI-AKI (AUC 0.753 vs. 0.604) with the optimal cutoff value for baseline NGAL to predict CI-AKI being 127 ng/mL (sensitivity of 68% and specificity of 68%, *p* < 0.001) (Figure 2).

Hazard ratios with corresponding 95% confidence intervals of the univariate and multivariate binary logistic regression for the development of CI-AKI are summarized in Table 2. In a multivariate logistic regression model, serum NGAL levels >127 ng/mL were independently associated with CI-AKI (HR 9.84, 95% CI: 1.96–40.3; *p* = 0.01). Other factors associated with CI-AKI included age, smoking history, and CRP.

## 4. Discussion

We demonstrated for the first time that, among CKD patients undergoing PCI, baseline NGAL levels were inversely correlated with eGFR. Furthermore, baseline NGAL performed better than eGFR as a marker of CI-AKI, with levels >127 ng/mL independently associated with CI-AKI.

CI-AKI following PCI is common, and even minute increases in sCr are associated with longer hospitalizations and unfavorable in-hospital and long-term outcomes. Among the various risk factors for CI-AKI, CKD ranks highly, with up to 50% of CKD patients developing this complication following contrast exposure [13,14]. Therefore, in these particularly vulnerable patients, simple surrogate markers corresponding both to baseline renal function and the development of CI-AKI are of importance.

NGAL, a 25-kDa protein covalently bound to gelatinase proteins in human neutrophils, was reported as an early marker of kidney tubular injury in various patient populations and hence was nicknamed “kidney troponin” [6,7,8,9]. Previous studies have shown the value of serum NGAL for the early diagnosis of CI-AKI in patients with normal renal function undergoing coronary procedures [17,18,19].

NGAL was originally isolated from the supernatant of activated human neutrophils but is also expressed at a low level in other human tissues, including the kidney [20]. NGAL is synthesized systemically in response to kidney damage followed by glomerular filtration and tubular uptake, and it could be produced locally by injured tubules. A third source of NGAL may be activated neutrophils/macrophages or inflamed vessels [20], which are frequently found in CKD. Previous reports demonstrated that serum NGAL is closely correlated with sCr, eGFR, and CRP and could thus serve as a marker of chronic impaired kidney function/kidney injury [21,22].

Worsening renal function may enhance overall inflammatory responses due to the decreased renal clearance of various factors directly or indirectly involved in the inflammatory cascade. In humans, deteriorating renal function may also affect the levels of additional inflammatory markers [23]. In the current cohort, patients developing CI-AKI demonstrated higher CRP levels than those who were not. Elevated CRP levels, reflecting inflammatory burden, were also shown to be associated with a higher risk of CI-AKI [24,25]. Thus, it is possible that elevated NGAL levels may reflect combined tubular injury and inflammatory response. We demonstrated an inverse correlation between serum NGAL level and calculated eGFR. In addition, according to the ROC curve, serum NGAL performed better than eGFR as a predictor of CI-AKI. Thus, among CKD patients, NGAL levels may well reflect both the severity of renal impairment while being independently associated with the risk of an acute deterioration following contrast administration.

Our findings suggest that the measurement of baseline plasma NGAL levels can assist in the stratification of those at high risk of further deterioration, as nearly 50% of patients with CKD demonstrated acute on chronic renal impairment. This type of patient profile is increasingly frequent among patients undergoing PCI, and, for these high-risk patients, preventive measures could help to limit renal damage and reduce mortality, as the available treatments for CI-AKI, once diagnosed, are limited. In these patients, in addition to pre-PCI hydration, one may consider deferring the use of drugs that may be potentially nephrotoxic (such as nonsteroidal anti-inflammatory drugs or angiotensin-converting enzyme inhibitors/angiotensin-II receptor blockers) and monitoring post-PCI serum NGAL for the early detection of early subclinical renal insult.

The current study has notable limitations. This is a single-center study with a modest sample size. In addition, our study is based on measurements of serum NGAL. The addition of urinary NGAL measurements to the analysis would have strengthened our conclusions; however, kits for measurements of urinary NGAL were not available for our institution. In patients with chronic renal insufficiency, urinary NGAL did not appear to have any predictive value for identifying those who would go on to develop CI-AKI [24,25]. Based on this data, it would seem that, in patients with chronic renal failure, plasma NGAL may be a better marker of CI-AKI than urinary NGAL. Finally, AKI diagnosis based on sCr may underestimate renal injury. The Acute Dialysis Quality Initiative (ADQI) reported a combination of kidney functional (sCr) and structural damage markers (new biomarkers including NGAL) to stratify patients with acute kidney damage [26]. Subclinical AKI can be diagnosed only using structural damage markers, such as NGAL, even when no change in sCr is observed (structural AKI). Indeed, among patients undergoing PCI, elevated NGAL levels with no concurrent sCr elevation were still associated with adverse clinical outcomes [26]. Finally, no data were present on patients’ baseline medications (such as angiotensin-converting enzyme/receptor blocker, diuretics, and other potential nephrotoxic drugs) which might have increased the risk of CI-AKI among CKD patients.

## 5. Conclusions

Among CKD patients, baseline NGAL levels may identify a population with a high risk for the development of CI-AKI. Further studies on larger populations are required to validate our reports and evaluate the potential utility of NGAL measurements in monitoring specific CKD-associated conditions.

## Figures and Tables

**Table 1:** Baseline characteristics and laboratory results for CKD patients with vs. without CI-AKI.

		CI-AKI Presence		
		No	Yes	*p*-Value
Total N (%)		50 (56.8)	38 (43.2)	
Age (years)	Mean (SD)	77.9 (10.9)	76.2 (9.1)	0.426
Gender	Female	11 (22.0)	10 (26.3)	0.801
	Male	39 (78.0)	28 (73.7)	
Diabetes mellitus	No	27 (54.0)	20 (52.6)	1.000
	Yes	23 (46.0)	18 (47.4)	
Body mass index	Median (IQR)	26.4 (5.0)	25.3 (2.7)	0.087
Hyperlipidemia	No	13 (26.0)	9 (23.7)	1.000
	Yes	37 (74.0)	29 (76.3)	
Family history of IHD	No	45 (90.0)	34 (89.5)	1.000
	Yes	5 (10.0)	4 (10.5)	
History of smoking	No	30 (60.0)	28 (73.7)	0.256
	Yes	20 (40.0)	10 (26.3)	
Hypertension	No	7 (14.0)	7 (18.4)	0.770
	Yes	43 (86.0)	31 (81.6)	
History of myocardial infarction	No	25 (50.0)	20 (52.6)	0.833
	Yes	25 (50.0)	18 (47.4)	
CAD severity (number of involved vessels)	0	8 (16.0)	5 (13.2)	0.231
	1	14 (28.0)	6 (15.8)	
	2	10 (20.0)	5 (13.2)	
	3	18 (36.0)	22 (57.9)	
LV ejection fraction, %	Median (IQR)	50.0 (18.8)	40.0 (15.0)	0.002
Reduced ejection fraction *	No	36 (72.0)	17 (44.7)	0.015
	Yes	14 (28.0)	21 (55.3)	
eGFR (mL/min/1.73 m^2^)	Median (IQR)	48.5 (20.8)	43.0 (18.9)	0.094
NGAL (ng/mL)	Median (IQR)	103.5 (56.4)	150.5 (77.8)	<0.001
C-reactive protein (mg/L)	Median (IQR)	4.3 (6.6)	14.3 (23.3)	<0.001
Hemoglobin (g/dL)	Median (IQR)	13.3 (2.0)	12.7 (3.3)	0.273
Troponin (×10^3^, mg/mL)	Median (IQR)	7.6 (40.3)	10.2 (193.4)	0.299
Contrast volume (mL)	Mean (SD)	113 ± 47	120 ± 48	0.194

SD, standard deviation; IHD, ischemic heart disease; CAD, coronary artery disease; LV, left ventricle; eGFR, estimated glomerular filtration rate; NGAL, neutrophil gelatinase-associated lipocalin; * defined as left ventricular ejection fraction ≤45%.

**Table 2:** Univariate and multivariate logistic regression models for the prediction of acute kidney injury.

		CI-AKI Presense			
		No	Yes	OR (Univariable)	OR (Multivariable)
Age (years)	Mean (SD)	77.9 (10.9)	76.2 (9.1)	0.98 (0.94–1.03, *p* = 0.433)	0.90 (0.82–0.98, *p* = 0.028)
Gender	Female	11 (52.4)	10 (47.6)	-	-
	Male	39 (58.2)	28 (41.8)	0.79 (0.29–2.14, *p* = 0.638)	0.43 (0.09–1.94, *p* = 0.279)
Diabetes mellitus	No	27 (57.4)	20 (42.6)	-	-
	Yes	23 (56.1)	18 (43.9)	1.06 (0.45–2.47, *p* = 0.899)	0.34 (0.07–1.44, *p* = 0.164)
Body mass index	Mean (SD)	27.5 (4.8)	25.8 (6.1)	0.94 (0.85–1.02, *p* = 0.169)	0.91 (0.76–1.04, *p* = 0.205)
Smoking history	No	30 (51.7)	28 (48.3)	-	-
	Yes	20 (66.7)	10 (33.3)	0.54 (0.21–1.32, *p* = 0.182)	0.21 (0.04–0.88, *p* = 0.042)
CAD (number of involved vessels)	0	8 (61.5)	5 (38.5)	-	-
	1	14 (70.0)	6 (30.0)	0.69 (0.15–3.06, *p* = 0.615)	0.75 (0.04–13.89, *p* = 0.843)
	2	10 (66.7)	5 (33.3)	0.80 (0.16–3.84, *p* = 0.778)	4.51 (0.25–99.48, *p* = 0.315)
	3	18 (45.0)	22 (55.0)	1.96 (0.55–7.47, *p* = 0.304)	6.71 (0.59–96.12, *p* = 0.136)
LV ejection fraction, %	Mean (SD)	48.5 (9.2)	41.6 (10.3)	0.93 (0.88–0.97, *p* = 0.003)	0.94 (0.88–1.01, *p* = 0.120)
eGFR (mL/min/1.73 m^2^)	Mean (SD)	47.1 (12.1)	42.5 (11.6)	0.97 (0.93–1.00, *p* = 0.081)	0.97 (0.90–1.04, *p* = 0.399)
Positive NGAL *	No	35 (74.5)	12 (25.5)	-	-
	Yes	15 (36.6)	26 (63.4)	5.06 (2.08–13.00, *p* = 0.001)	9.84 (1.96–40.33, *p* = 0.012)
Hemoglobin (g/dL)	Mean (SD)	13.2 (1.7)	12.6 (2.7)	0.88 (0.71–1.07, *p* = 0.197)	1.01 (0.74–1.40, *p* = 0.934)
C-reactive protein (mg/L)	Mean (SD)	10.3 (21.8)	18.7 (16.8)	1.03 (1.00–1.06, *p* = 0.079)	1.04 (1.01–1.08, *p* = 0.027)
Troponin (×10^3^, ng/dL)	Mean (SD)	42.5 (90.8)	1349.4 (7371.1)	1.00 (1.00–1.01, *p* = 0.110)	1.00 (1.00–1.01, *p* = 0.462)

OR, odds ratio; SD, standard deviation; CAD, coronary artery disease; LV, left ventricle; eGFR, estimated glomerular filtration rate; NGAL, neutrophil gelatinase-associated lipocalin; CRP, C-reactive protein; * defined as NGAL >127.

## Data Availability

Data available from the authors on request.
